# Inhibitory Effects of Angelica Polysaccharide on Activation of Mast Cells

**DOI:** 10.1155/2016/6063475

**Published:** 2016-04-20

**Authors:** Wei-An Mao, Yuan-Yuan Sun, Jing-Yi Mao, Li Wang, Jian Zhang, Jie Zhou, Khalid Rahman, Ying Ye

**Affiliations:** ^1^Department of Dermatology, Seventh People's Hospital, Shanghai University of Traditional Chinese Medicine, Shanghai 200137, China; ^2^Department of Graduate, Bengbu Medical College, Bengbu 233000, China; ^3^Yueyang Hospital, Shanghai University of Traditional Chinese Medicine, Shanghai 200437, China; ^4^School of Pharmacy and Biomolecular Sciences, Faculty of Science, Liverpool John Moores University, Liverpool L3 3AF, UK; ^5^Central Laboratory, Seventh People's Hospital, Shanghai University of Traditional Chinese Medicine, Shanghai 200137, China

## Abstract

This study was designed to investigate the inhibitory effects of Angelica polysaccharide (AP) on activation of mast cells and its possible molecular mechanism. In our study, we determined the proinflammatory cytokines and allergic mediators in anti-DNP IgE stimulated RBL-2H3 cells and found that AP (50, 100, and 200 *μ*g/mL) significantly decreased the release of histamine, *β*-hexosaminidase, leukotrienes C4 (LTC4), IL-1, IL-4, TNF-*α*, IL-6, and human monocyte chemotactic protein-1 (MCP-1/CCL2) (*p* < 0.05). In addition, Ca^2+^ entry was inhibited by treatment with AP. AP also downregulated the protein expressions of p-Fyn, p-Akt, p-P38, IL-4, TNF-*α*, and NF-*κ*B p65 in both Fyn gene upregulated and normal RBL-2H3 cells (*p* < 0.05). Collectively, our results showed that AP could inhibit the activation of mast cells via suppressing the releases of proinflammatory cytokines allergic mediators, Gab2/PI3-K/Akt and Fyn/Syk pathways.

## 1. Introduction

Allergic disorders, such as eczema, allergic rhinitis, and asthma, are generally considered as intractable diseases threatening people's health with an increasing prevalence in recent years [1, 2]. There is increasing evidence that mast cells play crucial roles in the development and pathogenesis of allergic diseases [3, 4]. In addition, allergic disorders are commonly caused by hypersensitive response to various allergens, such as proteins, pollen, chemicals, dust, and ultraviolet radiation [5]. Human's immune system would be sensitized after initial stimulation by an allergen. Thus, when rechallenged by the same allergen, the mast cells would be activated and degranulated; subsequently, various proinflammatory cytokines and allergic mediators would be released, leading to systemic allergic reactions [6, 7].

*Angelica sinensis* (Oliv.) Diels, belonging to the family of Apiaceae, is one of the well-known and commonly used traditional Chinese medicines. In traditional Chinese medicine theory,* A*.* sinensis *is a widely and commonly used drug for treating blood deficiency, inflammatory and gynecological diseases, and so forth. Current research indicates that* A*.* sinensis* is effective in the treatment of cardio- and cerebrovascular and immune nervous systems diseases, and so forth [8, 9]. In recent years, Angelica polysaccharide (AP) has been identified as one of the important and active components of* A*.* sinensis*. Increasing investigations have demonstrated that the AP possesses a wide range of pharmacological activities on the immune and circulatory system of humans including antitumor activity, immunoregulatory effect, radioprotective effect, and inhibition of platelet aggregation [10, 11].

As part of our continuing investigation on discovering candidate agents from TCMs, AP showed notable antiallergic effect* in vitro* in our preliminary experiment. Therefore, our present research was designed to systemically investigate the inhibitory effects of AP on activation of mast cells and its possible molecular mechanism, which would provide a scientific basis for the clinical use of AP to treat allergic disorders.

## 2. Materials and Methods

### 2.1. Chemicals and Reagents

Angelica polysaccharide (AP) was purchased from JRDUN Biotechnology Co. Ltd. (Shanghai, China); DMEM and fetal bovine serum (FBS) were purchased from Gibco. Co. (NY, USA); Cell Counting Kit-8 (CCK-8) was purchased from Dojindo Biochem (Shanghai, China); antidinitrophenol (DNP) IgE and DNP-HAS were purchased from Sigma-Aldrich (MO, USA); Fluo-3 AM reagents were purchased from Life Tech. Co. (CA, USA); rats histamine, IL-1, TNF-*α*, IL-6, LTC4, *β*-hexosaminidase, and MCP-1/CCL2 ELISA kits were purchased from the Boster Co. (Wuhan, China); p-Fyn and Fyn primary antibodies were purchased from Abcam Co. (Cambridge, UK); p-Akt, Akt, p-P38, P38, and NF-*κ*B p65 primary antibodies were purchased from CST Co. (MA, USA); TNF-*α*, IL-4, and GAPDH primary antibodies were purchased from Santa Cruz Biotech. (CA, USA); BCA protein kit and horseradish peroxidase- (HPR-) conjugated secondary antibodies were purchased from Beyotime Co. (Jiangsu, China); PVDF membrane was purchased from Millipore Biotech. (MA, USA).

### 2.2. Cell Culture and Cell Viability Assay

RBL-2H3 cells were purchased from the American Type Culture Collection (MD, USA) and were cultured in DMEM containing 10% (v/v) heat-inactivated FBS, 100 IU/mL penicillin, and 100 *μ*g/mL streptomycin at 37°C in a humidified atmosphere with 5% CO_2_.

Cell viability determination was carried out by using the CCK-8 assay [12]. Briefly, RBL-2H3 cells (5 × 10^4^/100 *μ*L) were seeded in 96-well plates and cultured at 37°C for 24 h. Then, 100 *μ*L serum-free DMEM containing 10% CCK-8 reagents (v/v) was added in each well, and cells were cultured for 1 h at 37°C. Subsequently, optical density (OD) values were determined at 450 nm by using a 96-well plate reader (DNM-9602, Pulang New technology, Beijing, China).

### 2.3. Degranulation Assay in RBL-2H3 Cells

RBL-2H3 cells (2 × 10^5^/well) were seeded in 24-well plates and stimulated with anti-DNPIgE (100 ng/mL) for 12 h. Then, the culture solution of RBL-2H3 cells was refreshed, and cells were treated with AP (50, 100, and 200 *μ*g/mL) and azelastine (used as positive drugs, 30 *μ*g/mL) for 1 h. The cells were then washed with Tyrode's buffer three times followed by incubation with DNP-HAS (20 ng/mL) for 30 and 120 min, respectively. (1) Then, for the cells incubated with DNP-HAS (20 ng/mL) for 30 min, the supernatant of the cell mixture was collected and the release of *β*-hexosaminidase, histamine, and LTC4 was determined by using commercial ELISA kits. In addition, the cells were also harvested for determining the releases of *β*-hexosaminidase and the inhibition of *β*-hexosaminidase release was calculated. (2) For the cells incubated with DNP-HAS (20 ng/mL) for 120 min, the supernatant of the cell mixture was collected, and IL-1, IL-4, TNF-*α*, and CCL2 were assayed by ELISA kits according to the instructions provided by the supplier [7].

### 2.4. Determination of Intracellular Ca^2+^ Concentrations

The concentration of Ca^2+^ was determined by using Fluo-3 AM Calcium Kits according to the manufacturer's instructions. Briefly, RBL-2H3 cells were seeded into the 6-well culture plate and treated with anti-DNP IgE. Subsequently, cells were incubated with 1 mL Fluo-3 AM for 1 h, and the fluorescent intensity was determined by using the flow cytometer (Accuri C6, BD, NJ, USA) [6].

### 2.5. Western Blot Assay

RBL-2H3 cells (2 × 10^5^/well) were seeded in 24-well plates and stimulated with anti-DNP IgE (100 ng/mL) for 12 h. Then, the culture solution of RBL-2H3 cells was refreshed, and cells were treated with AP (50, 100, and 200 *μ*g/mL) for 1 h and the cells were washed with Tyrode's buffer three times. Following this, cells were incubated with DNP-HAS (20 ng/mL) for 10 min and the total proteins were extracted, and their concentration was determined by BCA protein kit and 30 *μ*g total proteins were separated by sodium dodecyl sulfate- (SDS-) polyacrylamide gel electrophoresis (PAGE) and subsequently transferred to a PVDF membrane. The transferred protein PVDF membrane was probed with various primary antibodies, followed by incubation with HPR-conjugated secondary antibodies. Finally, chemiluminescence detection was used to visualize the target protein bands. To normalize protein loading, antibodies directed against GAPDH were used, and the proteins expression levels were expressed as a relative value to that of GAPDH.

### 2.6. Plasmid Construction and Transient Transfection

Fyn upregulated RBL-2H3 cells were constructed by the JRDUN Biotech. Co. (Shanghai, China). Briefly, the human Fyn gene was subcloned into a lentiviral vector [pCDNA3.1 (+)] to generate the lentiviral expression vector [pCDNA3.1 (+)-Fyn]. The recombinant lentiviruses were then produced by 293 T cells following the cotransfection of pCDNA3.1 (+)-Fyn. The resulting recombinant lentiviruses carrying Fyn were used to infect RBL-2H3 cells. The Fyn expression in untreated RBL-2H3 cells, cells treated with control vector (MOCK group), and Fyn gene overexpressed RBL-2H3 cells (Fyn-RBL-2H3) were detected by using real-time fluorogenic PCR (qRT-PCR) and western blotting assay.

### 2.7. Real-Time Fluorogenic PCR Assays

RBL-2H3 cells were harvested, and total RNA was extracted using Trizol reagent (Invitrogen, USA). Total RNA was used for cDNA synthesis of NF-*κ*B p65, TNF-*α*, Fyn, IL-4, and GAPDH by reverse transcription using qRT-PCR (ABI-7300, USA). All mRNA primers were designed by Premier 5.0 and synthesized by JRDun Biotech. (Shanghai, China). Primers used for the real-time PCR are shown in Table 1. Reverse transcription was performed according to the manufacturer's recommendation of the quantitative RT-PCR reaction kits (SYBR Green, Thermo Fisher Scientific, Shanghai, China).

### 2.8. Statistical Analyses

Data are presented as means ± standard deviation. Statistically significant differences were analyzed using two-tailed Student's *t*-test; *p* < 0.05 was considered to represent a statistically significant difference.

## 3. Results

### 3.1. Effects of AP on Degranulation in RBL-2H3 Cells Stimulated with Anti-DNP IgE

**Figure 1:**
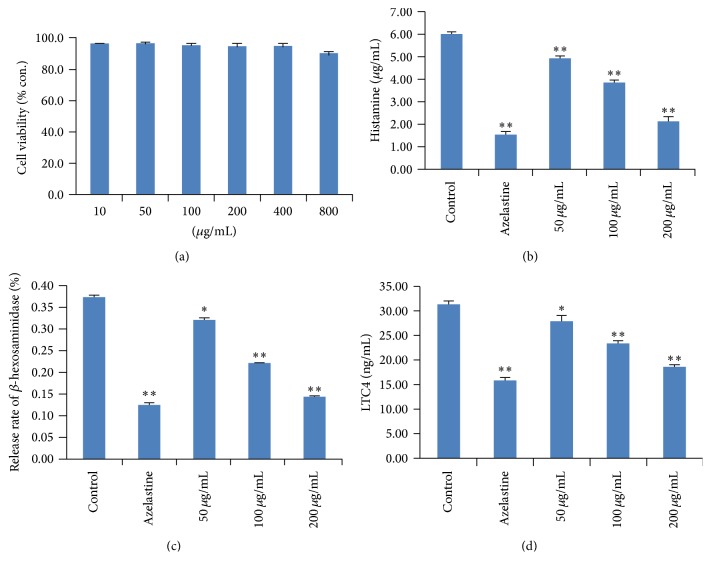
Effects of AP on degranulation in RBL-2H3 cells stimulated with anti-DNP IgE. (a) Cytotoxicity of AP on RBL-2H3 cells. (b) Effects of AP on histamine in RBL-2H3 cells. (c) Effects of AP on *β*-hexosaminidase in RBL-2H3 cells. (d) Effects of AP on LTC4 in RBL-2H3 cells. Azelastine was used as positive control. Data were represented as mean ± SD (*n* = 6), ^*⁎*^ *p* < 0.05, and ^*⁎⁎*^ *p* < 0.01, compared with control group.

As can be seen from Figure 1(a), cell viability assay showed no obvious cytotoxic effect of AP on RBL-2H3 cells within the concentrations tested (0–800 *μ*g/mL). Based on the results of cytotoxicity determination, the concentrations of 50, 100, and 200 *μ*g/mL without cytotoxicity were selected following the experiments. Furthermore, our results showed that AP at concentrations of 50, 100, and 200 *μ*g/mL possessed significant histamine suppressing activities compared to the control group, in a concentration-dependent manner (*p* < 0.01) (Figure 1(b)). Additionally, AP (50, 100, and 200 *μ*g/mL) also showed notable inhibitory effects on *β*-hexosaminidase and leukotrienes C4 (LTC4) in a concentration-dependent manner when compared to the control group (*p* < 0.05, *p* < 0.01, and *p* < 0.01, resp.) (Figures 1(c) and 1(d)).

**Figure 2:**
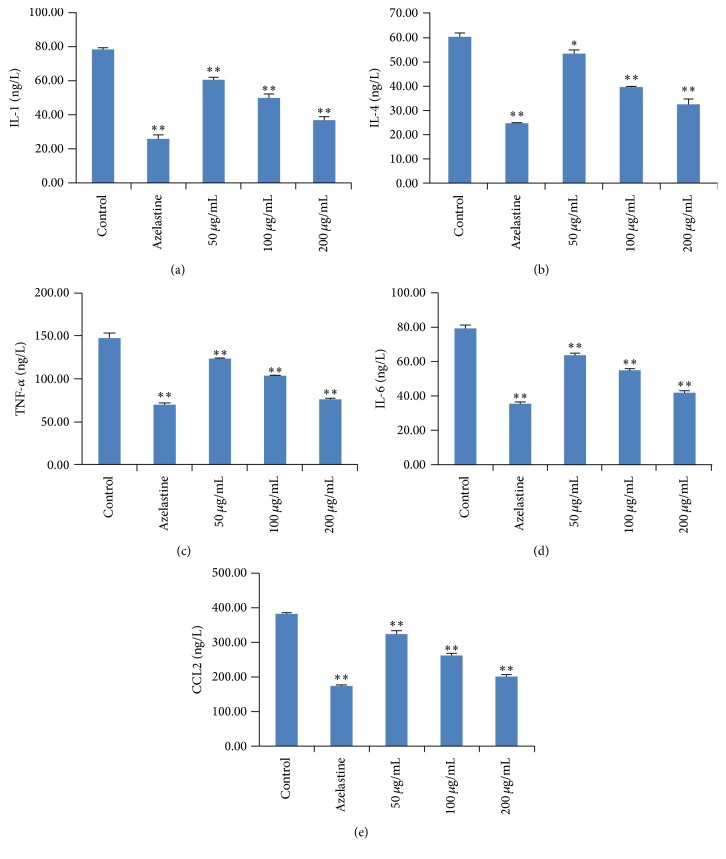
Effects of AP on the releases of IL-1 (a), IL-4 (b), TNF-*α* (c), IL-6 (d), and MCP-1/CCL2 (e) in RBL-2H3 cells stimulated with anti-DNP IgE. The azelastine was used as positive control. Data were represented as mean ± SD (*n* = 6), ^⁎^ *p* < 0.05, and ^⁎⁎^ *p* < 0.01, compared to control group.

Furthermore, compared to the control group, IL-1, TNF-*α*, IL-6, and human monocyte chemotactic protein-1 (MCP-1/CCL2) were also significantly inhibited by AP (50, 100, and 200 *μ*g/mL) with a concentration-dependent manner (*p* < 0.01) (Figure 2). Besides, AP (50, 100, and 200 *μ*g/mL) also significantly decreased the release of IL-4 (*p* < 0.05, *p* < 0.01, and *p* < 0.01, resp.), in a concentration-dependent manner.

### 3.2. Effects of AP on Ca^2+^ Entry in RBL-2H3 Cells Stimulated with Anti-DNP IgE

**Figure 3:**
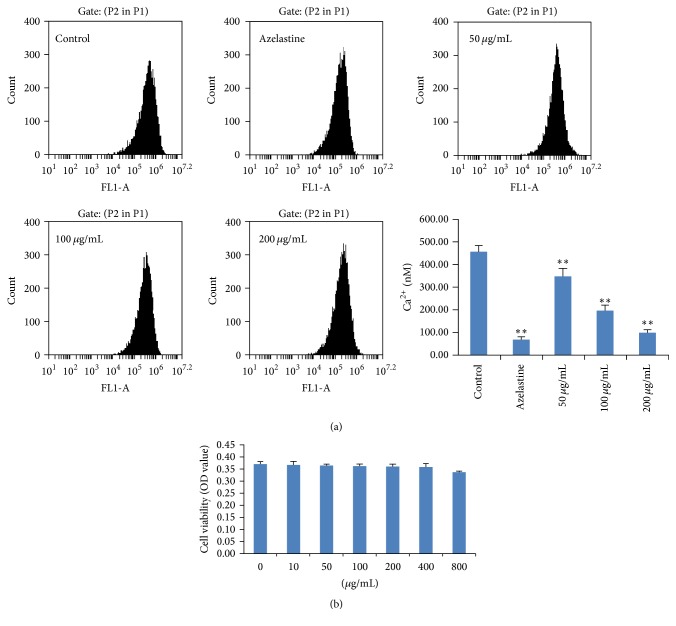
Effects of AP on Ca^2+^ entry in RBL-2H3 cells stimulated with anti-DNP IgE. The azelastine was used as positive control. Data are represented as mean ± SD (*n* = 6), ^⁎⁎^ *p* < 0.01, compared to control group.

In our present investigation, we also determined Ca^2+^ entry in RBL-2H3 cells induced by anti-DNP IgE by using Fluo-3-AM in conjunction with the FLIPR system. Our present results showed that AP could significantly decrease the Ca^2+^ influx in RBL-2H3 cells stimulated with anti-DNP IgE at the concentrations of 50, 100, and 200 *μ*g/mL (*p* < 0.01), compared to the control group (Figure 3). Besides, we can also find an obvious concentration-dependent manner for inhibiting Ca2+ influx in the present study.

### 3.3. Effects of AP on Protein Expressions of p-Fyn, Fyn, p-Akt, Akt, p-P38, P38, IL-4, TNF-*α*, and NF-*κ*B p65

**Figure 4:**
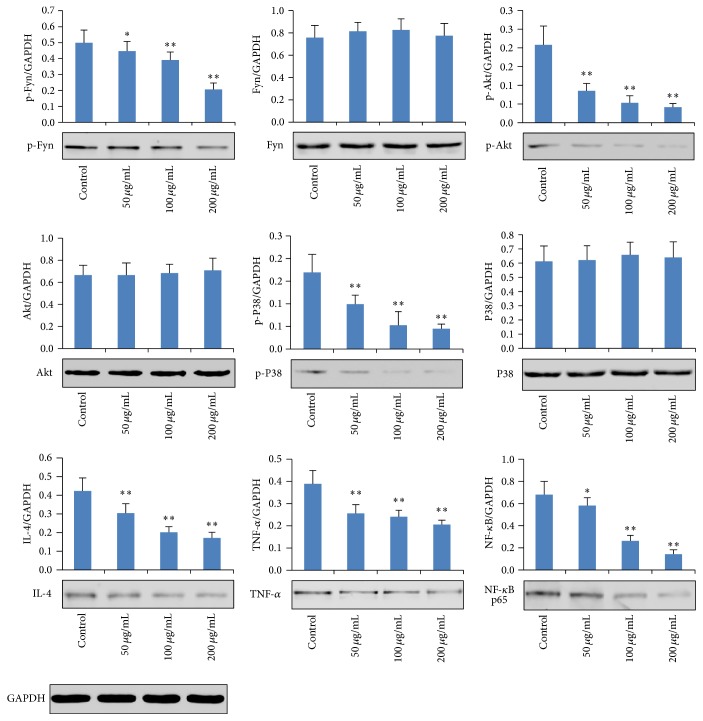
Effects of AP on protein expressions of p-Fyn, Fyn, p-Akt, Akt, p-P38, P38, IL-4, TNF-*α*, and NF-*κ*B p65. Data are represented as mean ± SD (*n* = 6), ^⁎^ *p* < 0.05, and ^⁎⁎^ *p* < 0.01, compared with control group.

Furthermore, by using western bolt assay, our study also investigates the protein expressions of p-Fyn, Fyn, p-Akt, Akt, p-P38, P38, IL-4, TNF-*α*, and NF-*κ*B in RBL-2H3 cells stimulated with anti-DNP IgE. The results indicate that the protein expressions of p-Akt, p-P38, IL-4, and TNF-*α* were significantly downregulated by treatment with AP (50, 100, and 200 *μ*g/mL) in a concentration-dependent manner (*p* < 0.01), when compared to the control group. Similarly, treatment with AP also downregulated the protein expressions of p-Fyn and NF-*κ*B p65 (50, 100, and 200 *μ*g/mL) (*p* < 0.05, *p* < 0.01, and *p* < 0.01, resp.), when compared to the control group. In contrast, no obvious difference was observed in the protein expressions of Fyn, Akt, and P38 (*p* > 0.05), compared to the control group (Figure 4).

### 3.4. Expression of Fyn in RBL-2H3 Cells after Transient Transfection

**Figure 5:**
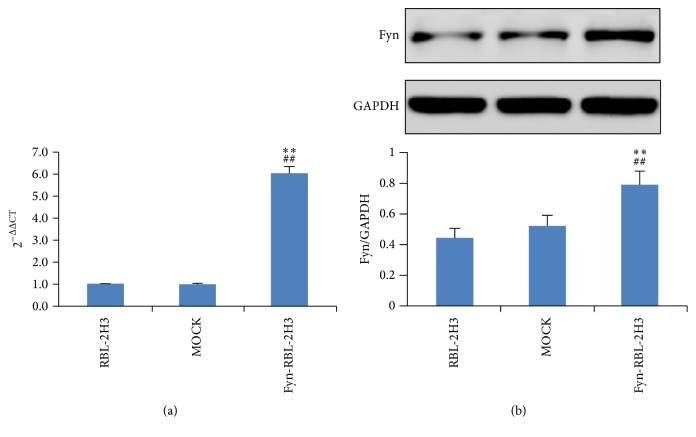
Expression of Fyn in RBL-2H3 cells after transient transfection. (a) mRNA expressions of Fyn determined by real-time PCR (qRT-PCR). (b) Protein expressions of Fyn determined by western blot assay. MOCK means the cells treated with control vector, and Fyn-RBL-2H3 means Fyn gene upregulated RBL-2H3 cells, ^⁎⁎^ *p* < 0.01, compared to untreated RBL-2H3 cells, and ^##^ *p* < 0.01, compared to MOCK group.

In order to confirm the importance of Fyn gene in the activation of mast cells, the Fyn upregulated RBL-2H3 cells were constructed by transient transfection. As can be seen from Figure 5, after transient transfection, the Fyn gene was significantly upregulated in the RBL-2H3 cells (*p* < 0.01), compared to both untreated RBL-2H3 and MOCK groups (Figure 5(a)). Furthermore, the results of our western blotting assay also demonstrated that Fyn protein was upregulated after transient transfection (Figure 5(b)).

### 3.5. Effects of AP on mRNA Expressions of NF-*κ*B p65, IL-4, and TNF-*α* in Upregulated RBL-2H3 Cells Stimulated with Anti-DNP IgE

**Figure 6:**
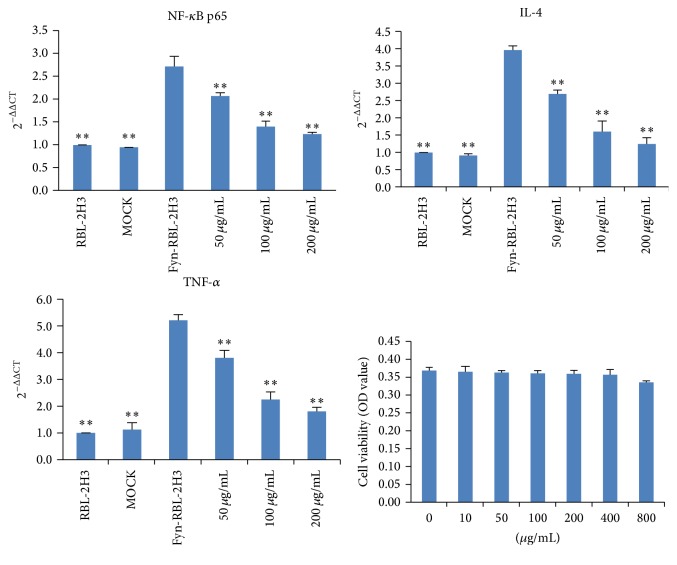
Effects of AP on mRNA expressions of NF-*κ*B p65, IL-4, and TNF-*α* in upregulated RBL-2H3 cells stimulated with anti-DNP IgE. MOCK means the cells treated with control vector, and Fyn-RBL-2H3 means Fyn gene upregulated RBL-2H3 cells, ^⁎⁎^ *p* < 0.01, compared to Fyn-RBL-2H3 cells.

After the Fyn upregulated RBL-2H3 cells were established, we determined the mRNA expressions of NF-*κ*B p65, IL-4, and TNF-*α* in upregulated RBL-2H3 cells induced by anti-DNP IgE. Our results indicated that the mRNA expressions of NF-*κ*B p65, IL-4, and TNF-*α* genes in RBL-2H3 cells were significantly increased in both untreated RBL-2H3 cells (*p* < 0.01) and MOCK groups (*p* < 0.01). However, treatment with AP (50, 100, and 200 *μ*g/mL) reversed these increased mRNA expressions of NF-*κ*B p65 (*p* < 0.01), IL-4 (*p* < 0.01), and TNF-*α* (*p* < 0.01), in a concentration-dependent manner compared with the Fyn upregulated RBL-2H3 cells. In addition, our results also demonstrated that no obvious antiproliferation effect of AP (0–800 *μ*g/mL) was found in the growth of Fyn upregulated RBL-2H3 cells (Figure 6).

### 3.6. Effects of AP on Protein Expressions of p-Fyn, Fyn, p-Akt, Akt, p-P38, P38, IL-4, TNF-*α*, and NF-*κ*B p65 in Upregulated RBL-2H3 Cells Stimulated with Anti-DNP IgE

**Figure 7:**
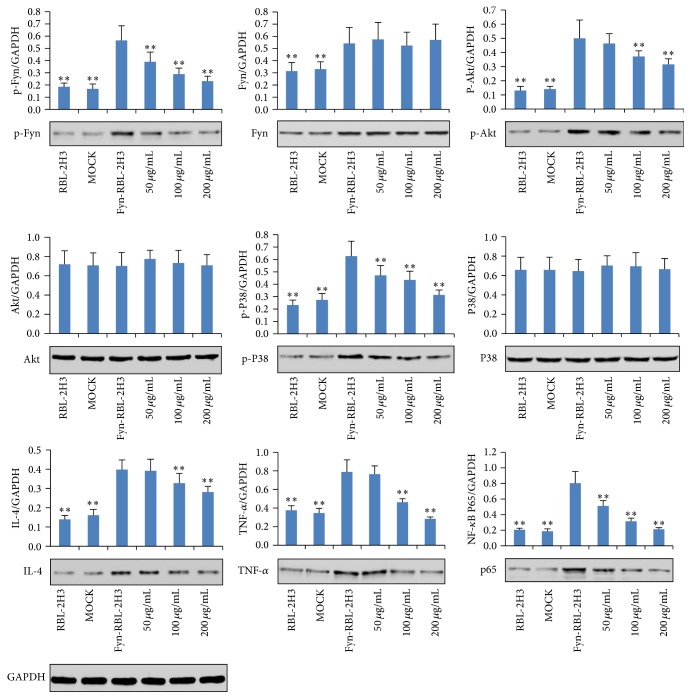
Effects of AP on protein expressions of p-Fyn, Fyn, p-Akt, Akt, p-P38, P38, IL-4, TNF-*α*, and NF-*κ*B p65 in upregulated RBL-2H3 cells stimulated with anti-DNP IgE. MOCK means the cells treated with control vector, and Fyn-RBL-2H3 means Fyn gene upregulated RBL-2H3 cells, ^⁎⁎^ *p* < 0.01, compared to Fyn-RBL-2H3 cells.

Furthermore, after treatment with AP (50, 100, and 200 *μ*g/mL), we determined the protein expressions of p-Fyn, Fyn, p-Akt, Akt, p-P38, P38, IL-4, TNF-*α*, and NF-*κ*B p65 in upregulated RBL-2H3 cells stimulated with anti-DNP IgE. As can be seen from Figure 7, the p-Fyn, Fyn, p-Akt, p-P38, IL-4, TNF-*α*, and NF-*κ*B p65 were upregulated (*p* < 0.01), compared with untreated RBL-2H3 cells. However, after treatment with AP (50, 100, and 200 *μ*g/mL), the expressions of p-Fyn, p-P38, and NF-*κ*B p65 were downregulated significantly (*p* < 0.01) in a concentration-dependent manner, compared to the Fyn-RBL-2H3 cells. In addition, the AP (100 and 200 *μ*g/mL) decreased the upregulated expressions of p-Akt, IL-4, and TNF-*α* (*p* < 0.01), when compared to the Fyn-RBL-2H3 cells.

## 4. Discussion

Eczema, one of the stubborn skin diseases with increasing prevalence, is closely correlated to the immune functions of human being. Currently, natural herbal medicines, such as TCMs, have aroused considerable interest due to their low toxicity and reliable therapeutic effects [12, 13]. Interestingly,* A*.* sinensis *is one of the most commonly used herbal medicines for treating eczema in China [14, 15]. However, no systemic investigation reporting the active substances and their therapeutic effect on eczema has been conducted. To the best of our knowledge, this is the first systemic investigation regarding inhibitory effect of Angelica polysaccharide (AP) on activation of mast cells and its possible molecular mechanism. In our present research, the AP showed significant inhibitory effect against the activation of RBL-2H3 cells. In addition, our present results also indicated that downregulating Fyn gene might be a possible molecular mechanism for responding to the activity of AP.

Previous researches reported that, after sensitization by various allergens, the mast cells would be activated and respond via degranulation [7, 16]. Subsequently, several of proinflammatory cytokines and allergic mediators are released, leading to immune response. Histamine and *β*-hexosaminidase are commonly considered as the notable markers in degranulation of mast cells [17]. Previous reports have demonstrated that they would be elevated in plasma or tissues in various allergic diseases. LTC4, IL-6, IL-1, MCP-1/CCL2, and TNF-*α* play important roles in the development of allergic diseases, and their release and synthesis could be increased in various allergic diseases [18, 19]. In our present study, we used the anti-DNP IgE, a commonly used allergen, to sensitize the RBL-2H3 cells. Then, we determined the releases of histamine, *β*-hexosaminidase, LTC4, and MCP-1/CCL2 in RBL-2H3 cells. Besides, mast cell degranulation and histamine production are Ca^2+^ dependent, and Ca^2+^ entry activates the degranulation of mast cells [7, 20]. Our present results showed that AP possessed significantly inhibitory effects on releases of proinflammatory cytokines, allergic mediators, and Ca^2+^ entry in RBL-2H3 cells stimulated with anti-DNP IgE, indicating that AP could effectively inhibit the degranulation and activation of mast cells. Alleviating the inflammatory reactions would be beneficial for controlling the allergic symptoms. Our results also revealed that AP treatment could inhibit the expressions of some crucial inflammatory pathway cytokines including IL-1, IL-6, TNF-*α*, and NF-*κ*B p65.

Fyn is a crucial signaling molecule for activation of mast cells stimulated by various antigens. Increasing reports have demonstrated that Gab2/PI3-K/Akt and Fyn/Syk pathway plays an essential role in the development of allergic diseases [21, 22]. Interestingly, in our present study, we also found that AP could downregulate phosphorylated Fyn in anti-DNP IgE stimulated RBL-2H3 cells. Furthermore, the phosphorylated downstream signaling molecules Gab2/PI3-K/Akt and Fyn/Syk pathway in anti-DNP IgE stimulated RBL-2H3 cells, including p-Akt and p-P38, were also downregulated. Thus, we proposed that the Fyn might be a potential molecular mechanism of AP for treating allergic diseases. To confirm our hypothesis, the gene upregulated RBL-2H3 cells were constructed by transient transfection. Importantly, similar results were also obtained in the Fyn upregulated RBL-2H3 cells stimulated with anti-DNP IgE. Our results showed that the AP treatment could downregulate the related cytokines and proteins in inflammatory pathway and essential proteins in Gab2/PI3-K/Akt and Fyn/Syk pathways.

## 5. Conclusions

In conclusion, our present investigation demonstrated that AP could inhibit the releases of proinflammatory cytokines and allergic mediators. In addition, our results also demonstrated that AP downregulated the related cytokines and proteins in inflammatory pathway and essential proteins in Gab2/PI3-K/Akt and Fyn/Syk pathways. Collectively, our results suggested that AP could inhibit the activation of mast cells.

## Figures and Tables

**Table 1:** Primers used in our real-time PCR experiment.

Genes	Sequences
NF-*κ*B p65	F: 5′ AGACCTGGAGCAAGCCATTAG 3′
	R: 5′ CGGACCGCATTCAAGTCATAG 3′

TNF-*α*	F: 5′ TGGCGTGTTCATCCGTTC 3′
	R: 5′ CTACTTCAGCGTCTCGTGTG 3′

Fyn	F: 5′ ACCACCAAAGGTGCCTACTC 3′
	R: 5′ ATGTAGTACCCGCCGTTGTC 3′

IL-4	F: 5′ CCTTGCTGTCACCCTGTTC 3′
	R: 5′ CTCGTTCTCCGTGGTGTTC 3′

GAPDH	F: 5′ GTCGGTGTGAACGGATTTG 3′
	R: 5′ TCCCATTCTCAGCCTTGAC 3′
